# Alterations in IL-6/STAT3 Signaling by Korean Mistletoe Lectin Regulate the Self-Renewal Activity of Placenta-Derived Mesenchymal Stem Cells

**DOI:** 10.3390/nu11112604

**Published:** 2019-10-30

**Authors:** Gi Dae Kim, Jong Ho Choi, Seung Mook Lim, Ji Hye Jun, Ji Wook Moon, Gi Jin Kim

**Affiliations:** 1 Department of Food and Nutrition, Kyungnam University, Gyeongsangnam-do 51767, Korea; gidaekim@kyungnam.ac.kr; 2 Department of Oral Pathology, College of Dentistry, Gangneung-Wonju National University, Gangneung-si 25457, Korea; jhchoi@gwnu.ac.kr; 3 Department of Biomedical Science, CHA University, Seongnam-si 13488, Korea; lsmook17@naver.com (S.M.L.); jihyejun1015@gmail.com (J.H.J.); 4 Department of Pathology, Korea University College of Medicine, Seoul 02841, Korea; Mjw6132@korea.ac.kr

**Keywords:** mesenchymal stem cell, mistletoe lectin, VCA, self-renewal, IL-6, methylation

## Abstract

Korean mistletoe (*Viscum album* L. var. *coloratum*) lectin (VCA) is known as an anticancer drug. However, it is not clear whether VCA affects the self-renewal activity of mesenchymal stem cells (MSCs). Therefore, the objectives of this study were to analyze the effect of VCA on the proliferation of MSCs and expression of stemness markers. We also evaluated the usefulness of placenta-derived MSCs (PD-MSCs) as a screening tool. VCA was stably administered to MSCs, and analyzed self-renewal activities. The effect of IL-6 signaling on MSC proliferation was explored by quantitative methylation-specific PCR (qMSP) and western blot analysis. Compared with the control condition, low concentrations of VCA (10 pg/mL) induced an increase in the self-renewal activity of MSCs. Interestingly, a low concentration of VCA promoted IL-6 signaling in PD-MSCs through altered IL-6/STAT3 gene methylation. Furthermore, inhibition of IL-6 expression in PD-MSCs using an anti-IL-6 antibody caused a decrease in their self-renewal activity through IL-6/STAT3 signaling by altering IL-6/STAT3 gene methylation. These findings provide helpful data for understanding the mechanism of MSC self-renewal via VCA and show that VCA may be useful as a functional natural product for developing efficient therapies using placenta-derived stem cells.

## 1. Introduction

The mesenchymal stem cells (MSCs) isolated from various adult tissues are used in cell therapy for degenerative disease because these cells have the potential to differentiate into cells of the mesenchymal lineage (e.g., chondrocytes, adipocytes and osteoblasts) [1] and exert a therapeutic effect [2]. Additionally, MSCs have immunosuppressive properties, a critical factor in cell therapy, through their regulation of the responses of natural killer cells, dendritic cells, B-cells and T-cells when engrafted into damaged tissues or organs [3,4,5]. However, the limited self-renewal activity of MSCs, which contrasts with the unlimited self-renewal of embryonic stem cells (ESCs), is one hurdle for cell therapies that use MSCs. Recently, placenta-derived mesenchymal stem cells (PD-MSCs) were highlighted as alternative cell sources of bone marrow-derived MSCs (BM-MSCs) because their self-renewal activity is more powerful than that of BM-MSCs [6]. In general, the self-renewal and/or differentiation potential of stem cell is not well understood because the pluripotency of adult stem cells, including MSCs, depends on the age of the donor. In contrast, PD-MSCs are consistently maintained from the placenta after full-term birth at the same starting point [6,7,8], though there are still obstacles to enhancing the limited self-renewal of PD-MSCs.

For this reason, many researchers have investigated strategies for overcoming the limited self-renewal ability of MSCs using various cultivation approaches [9,10]. Although advanced cultivation methods using supplements, such as fibroblast growth factor (FGF)-2, platelet-derived growth factor and epidermal growth factor (EGF), have been introduced by several groups [11,12], it is difficult to obtain a sufficient number of MSCs for clinical application. Additionally, the conventional cultivation of MSCs is a disadvantage because of the cost associated with the use of several growth factors to enhance the MSC growth rate [13]. Recently, an alternative MSC cultivation method using natural products was reported to be able to overcome the limitations of conventional cultivation [14,15]. Li X et al. reported that the volatile oil of *Piper longum* enhances expression of markers related to the proliferation of MSCs [16]. Additionally, the extract of *Cissus quadrangularis* (300 µg/mL) stimulates the proliferation of BM-MSCs and increases the differentiation potential into osteoblasts [17]. However, the modes of action involved remain unclear.

Korean mistletoes (*Viscum album* L. var. *coloratum*) lectin (VCA), which is known as an anticancer drug, has multiple functions in several cell types depending on the application. For example, VCA increased expression of apoptosis-related molecules such as tumor necrosis factor-alpha (TNF-α), caspase-2, caspase-3, caspase-8 and caspase-9 and decreased antiapoptosis molecules such as receptor-interacting protein (RIP), NF-kB and X-linked inhibitor of apoptosis protein (XIAP) [18,19,20]. In addition, VCA exerts an immunomodulatory effect on cells related to the immune system, including lymphocytes, NK cells and macrophages, through the TLR4 signaling pathway [21,22,23]. Furthermore, we reported that a low concentration (1–10 pg/mL) of VCA induces the proliferation and invasion ability of trophoblasts through Akt signaling [24] and that VCA regulates the self-renewal of PD-MSCs via autophagic mechanisms [25]. Nonetheless, the effect of VCA on the self-renewal of MSCs remains unclear.

In general, the self-renewal activity of stem cells is regulated by cell-intrinsic and cell-extrinsic signals, including epigenetic regulation [26,27]. Among cell-intrinsic factors, Oct4, Sox2 and Nanog, which are representative stemness markers, regulate the self-renewal and differentiation of stem cells [28,29]. For example, overexpression of Oct4 and Sox2 increases the proliferation of BM-MSCs [30]; moreover, downregulation of Oct4 by doxycycline, an inhibitor of cell cycle progression in G0/G1, decreases the proliferation of stem cells [31]. Coronado D et al. showed that abnormal regulation of Cyclin E is involved in the balance between the self-renewal and differentiation of stem cells [32]. Therefore, it is important to correctly control the cell cycle to maintain the self-renewal activity of stem cells.

Interleukin-6 (IL-6) is a well-known multifunctional cytokine that is a critical component of the Janus kinase (JAK)/signal transducer and activator of transcription (STAT3) pathway in several cell types [33]. IL-6 regulates cell cycle-related factors such as p53, p21 and epidermal growth factor receptor (EGFR) by modulating gene expression via epigenetic mechanisms [34,35]. Additionally, JAK/STAT3-mediated IL-6 phosphorylates mitogen-activated protein kinase (MAPK) and the Akt pathway, a major pathway in cancer proliferation [36]. Several studies have shown that increased expression and secretion of IL-6 contribute to tumor growth and metastatic spread in vivo and in vitro [37,38,39,40], as regulated by DNA methylation/hypermethylation in several types of cancer [41,42]. Recently, Choi B et al. reported that increased IL-6 induced by Notch signaling promotes the self-renewal maintenance of human CD34+ cord blood cells via activation of the JAK/PI3K/STAT3 pathway [43]. Additionally, expression of IL-6 is increased in MSCs, especially during proliferation [44]. However, the roles of IL-6 in the self-renewal activity of MSCs are unknown.

Therefore, the objectives of this study were to analyze expression of stemness markers (e.g., Oct4, Nanog, and Sox2) in PD-MSCs cultured with VCA, to analyze IL-6/STAT3 signaling in PD-MSCs following VCA treatment, and to evaluate the methylation pattern of IL-6/STAT3 genes in PD-MSCs following VCA treatment. Furthermore, we demonstrate the usefulness of PD-MSCs as a tool for screening functional natural products.

## 2. Materials and Methods

### 2.1. Cell Line and Cell Culture

Normal placentas were collected from term pregnancies (≥37 gestational weeks). No medical, obstetrical or surgical complications occurred. Placenta-derived mesenchymal stem cells (PD-MSCs) were isolated from normal term placentas as described previously [45]. In particular, they were isolated from the chorionic plate of the placenta and cultivated in alpha-modified Eagle’s medium (α-MEM; Gibco, Waltham, MD, USA) supplemented with 10% fetal bovine serum (FBS; Gibco), 100 U/mL penicillin/streptomycin (Pen/Strep; Gibco), 25 ng/mL fibroblast growth factor 4 (FGF4; Peprotech, Rocky Hill, NJ, USA), and 1 µg/mL heparin (Sigma, Saint Louis, MO, USA). The cells were used within 7~9 passages and treated with several concentrations of VCA (0–10,000 pg/mL) for 48 h. The extracts of VCA were provided by Park Won Bong (Seoul Women’s University, Seoul, Korea) [46]. The collection of human placentas and their use were approved by the Institute Review Board (IRB) of CHA General Hospital, Seoul, Korea (IRB no. 07-18).

### 2.2. Cell Growth Assays

To measure the growth of PD-MSCs, cells were plated at 4 × 10^4^ cells per plate in 60-mm dishes and counted at 24 h, 48 h, 72 h, 96 h and 120 h after seeding. The cells were then digested using trypsin (Invitrogen, Pleasanton, CA, USA), resuspended in 1 mL of culture medium and counted using a hemocytometer. Next, 0.2% trypan blue was added to the cell suspension to exclude nonviable cells from the counting. All experiments were performed in two separate replicates.

### 2.3. Differentiation into Mesodermal Lineages

To analyze the potential for differentiation into mesodermal lineages, PD-MSCs were plated at a density of 2 × 10^4^ cells/plate in 35-mm dishes and cultivated in α-MEM supplemented with 10% FBS, 100 U/mL Pen/Strep, 25 ng/mL FGF4, and 1 µg/mL heparin for 24 h. Adipogenic or osteogenic differentiation was induced using the following specific media: StemPro Adipogenesis Differentiation Kit (Gibco) or StemPro Osteogenesis Differentiation Kit (Gibco). The induction medium was replaced every 3–4 days until day 21. Oil-red O staining was then performed. To analyze the lipid drops in differentiated PD-MSCs, the cells were fixed in 4% paraformaldehyde (PFA) for 20 min, rinsed in 3% isopropanol and stained in 0.5% Oil Red O solution (Sigma) for 1 h 30 min. The cell nuclei were then stained with Mayer’s hematoxylin (Sigma) for 1 min. The lipid droplets in differentiated cells were visualized using an inverted microscope (Magnificent 100×). Von Kossa staining was performed, whereby osteogenic-induced cells were rinsed twice in PBS and fixed in 4% PFA for 20 min. The cells were washed in PBS and then stained using silver nitrate (Sigma) under light for 1 h. The calcium deposits in the differentiated cells were visualized using an inverted microscope (Magnificent 100×).

### 2.4. Differentiation into Endodermal Lineages

To analyze the potential of PD-MSCs to differentiate into hepatocytes, we used four step-induction protocols. A total of 2 × 10^4^ cells were seeded in 0.5% collagen type I-coated 60-mm dishes and cultivated in culture medium (60% DMEM-LG:40% MCDB201 with 2% FBS and 1% P/S) for 24 h and in conditioned medium (60% DMEM-LG:40% MCDB201 with 50 µg/mL EGF, 50 µg/mL lbFGF, 5 µg/mL BMP-4, and 1% P/S) for 48 h. The culture medium was then replaced with an early induction medium (60% DMEM-LG:40% MCDB201 with 2% FBS, 50 µg/mL FGF, 10 µg/mL HGF, and 1% P/S) for 7 days, after which the culture medium was replaced with maturation medium (60% DMEM-LG:40% MCDB20 with 10 µg/mL oncostatin M and 1 mM/mL DEXA) for 14 days. The early induction and maturation media were replaced every 3–4 days. An indocyanine green (ICG) uptake assay was then performed. The cells were washed twice in PBS, added to dishes containing 1 mg/mL ICG, and incubated at 37 °C for 1 h 30 min. The cells were then rinsed three times with PBS, and the dishes were refilled with maturation medium. Uptake of ICG was assessed using an inverted microscope (Magnificent 100×). Periodic acid-Schiff (PAS) staining was performed to analyze glycogen stores in hepatogenic-induced cells. The cells were fixed in 4% formaldehyde for 20 min and oxidized in 1% periodic acid for 10 min, after which the cells were washed using D. W and incubated in Schiff’s reagent for 15 min. The cells were stained with Mayer’s hematoxylin (Sigma) for 1 min and visualized using an inverted microscope (Magnificent 100×).

### 2.5. MTT Assay

The 3-(4,5-dimethylyhiazol-2-yl)-2,5-diphenyl tetrazolium bromide (MTT) assay was carried out to analyze the effect of VCA on the proliferation ability of PD-MSCs and BM-MSCs. PD-MSCs and BM-MSCs were seeded at 2.0 × 10^3^ cells per well on a 96-well plate (Thermo Scientific, Waltham, MA, USA) and cultivated at 37 °C in an incubator with a humidified atmosphere of 5% CO_2_. After 24 h, the cells were treated with VCA (0–10,000 pg/mL) for 48 h; 0.5 mg/mL MTT (Sigma) was added to the VCA-treated cells for 3 h, and then 200 µL of dimethyl sulfoxide (DMSO) (Sigma) was added for 10 min. The resulting formazan was dissolved in DMSO, and absorbance was detected at 570 nm with a VersaMax enzyme-linked immunosorbent assay (ELISA) microplate reader (Molecular Devices, San Jose, CA, USA). Experiments were performed in triplicate.

### 2.6. Neutralization of IL-6

To analyze the effect of IL-6 on the proliferation of PD-MSCs induced by VCA, 2.0 × 10^3^ or 0.75 × 10^6^ cells were cultured in 96-well plates (Thermo Scientific) or 100-mm dishes (Thermo Scientific) for 24 h and cocultured for 48 h with or without 4 µg/mL of the neutralizing antibody, anti-IL-6 (Peprotech), in culture medium supplemented with several concentrations of VCA (0–10 pg/mL).

### 2.7. RT-PCR and qRT-PCR

Total RNA was extracted from cells with Trizol reagent (Invitrogen); 250 µg of cDNA was synthesized using Superscript III RNase H reverse transcriptase (Invitrogen) according to the manufacturer’s protocol. The total RNA of each sample was examined by 1% agarose gel electrophoresis. The mRNA levels of target genes were determined by reverse transcription polymerase chain reaction (RT-PCR) using h-Taq DNA polymerase (Solgent, Seoul, Korea) and by quantitative real-time polymerase chain reaction (qRT-PCR) using SYBR Green master mix (Hoffmann-La Roche, Basel, Switzerland) according to the manufacturers’ instructions. The specific primer sequences are displayed in Supplementary Table S1. The mRNA amplification conditions for qRT-PCR were precooling at 95 °C for 5 min, followed by 40 cycles at 95 °C for 5 s and 60 °C for 30 s. Reactions were performed in triplicate and analyzed using EXICYCLER^TM^ 96 (Bioneer, Daejeon, Korea). Relative expression levels were calculated using the ∆∆Ct method. GAPDH and 18S were used as internal controls for normalization of the qRT-PCR results.

### 2.8. FACS Analysis

Expression of surface markers associated with mesenchymal stem cells, such as CD13, CD34, CD44, CD45, CD95, CD105, HLA-DR and HLA-ABC, was evaluated by flow cytometry. Cultured cells at passage number 9 were detached from dishes with nonenzymatic cell dissociation solution (Sigma) and washed twice in PBS at room temperature. Cell pellets containing 2.5 × 10^5^ cells were resuspended in 0.1 mM PBS and then incubated with isotype control IgG or specific antibodies, including CD-34, CD-44 and 90-PE (BD Biosciences, San Jose, CA, USA), CD-95-APC (BD Biosciences), CD-45-FITC, HLA-ABC-FITC and HLA-DR-FITC (BD Biosciences). A total of 1 × 10^4^ cells were analyzed using a FACScan flow cytometer (BD Biosciences) and quantified with CellQuest Pro Software (BD Biosciences).

### 2.9. Genomic DNA Extraction and Sodium Bisulfite DNA Modification

Genomic DNA was extracted from PD-MSCs and BM-MSCs using QIAamp DNA Mini Kit (Qiagen, Hilden, Germany). MSCs were ground with 3-mm diameter puncher and then lysed with 700 μL of lysis buffer supplemented with 20 μg/mL Labo Pass protease K (Cosmo Gene Tech., Seoul, Korea), 20 mM Tris∙HCl (pH 8.0), 5 mM EDTA (pH 8.0), 400 mM NaCl and 1% sodium dodecyl sulfate (SDS) solution (Sigma) at 42 °C overnight, followed by purification with phenol/chloroform. Genomic DNA was eluted with 100 μL of water and quantified using a NanoDrop ND-100 device (Thermo Fisher Scientific). Two micrograms of genomic DNA in a volume of 20 μL of RNase-free water was subjected to bisulfite conversion using the EpiTect^®^ fast DNA bisulfite kit (Qiagen) according to the manufacturer’s protocol. The reactions were performed by mixing the DNA-containing bisulfite, 85 μL of mix and 35 μL of DNA protection buffer in a PCR tube at room temperature.

### 2.10. qMSP

To quantify the methylation status of IL-6 and STAT3 genes in PD-MSCs and BM-MSCs following treatment with VCA at various concentrations, bisulfite-modified DNA was subjected to quantitative real-time PCR using a 7000 HT Real-Time PCR System (Applied Biosystems, Foster City, CA, USA) according to the manufacturer’s instructions. Methylation-specific primers were designed using MethPrimer software (http://www.urogene.org/methprimer); the primer sequences used for qMSP are shown in Supplementary Table S2. A sample of 30 ng of bisulfite-converted DNA template was amplified using the 2X Maxima^®^ SYBR Green/ROX qPCR master mixes (Thermo Fisher Scientific) and 250 nM of each primer under the following conditions: initial incubation at 50 °C for 2 min, denaturation at 95 °C for 10 min, and 45 cycles of 15 s at 95 °C and 1 min at 60 °C. After PCR amplification, relative quantification of the amplified IL-6 and STAT3 levels was performed by measuring the Ct values of the target and internal control genes against serial dilutions of bisulfite-modified DNA at known concentrations. DNA treated with CpG methyltransferase (M. SssI; New England Biolabs, Ipswich, MA, USA) was used as a positive control; β-actin was used as an internal control. Each DNA sample was analyzed in duplicate.

### 2.11. Western Blot Analysis

Cells were lysed by scraping them into RIPA buffer (Sigma) containing protease inhibitor cocktail (Roche Diagnostics GmbH, Mannheim, Germany) and phosphatase inhibitor cocktail II (A.G. Scientific Inc., San Diego, CA, USA). Equal concentrations of protein (60 µg) lysates were separated by SDS polyacrylamide gel electrophoresis and blotted onto a polyvinylidene difluoride membrane (PVDF; Bio-Rad Laboratories, Inc., Hercules, CA, USA). The membranes were blocked with 8% skim milk (BD Biosciences) in PBS containing 0.05% Tween 20 (PBS-T, Sigma) or 5% BSA (AMRESCO, Cleveland, OH, USA) in TBS containing 0.1% Tween 20 (TBS-T, Sigma) for 1 h at room temperature and incubated with specific primary antibodies overnight at 4 °C. Primary antibodies against IL-6 (1:1000, Abcam, Cambridge, MA, USA), p-STAT3 and GAPDH (1:1000, Cell Signaling Technology, Danvers, MA, USA), t-STAT3 (1:1000, Sigma), and Cyclin D (1:1000, Ab Frontier, Seoul, Korea) were used. The membranes were washed and incubated with horseradish peroxidase-conjugated anti-rabbit immunoglobulin G (IgG; 1:25,000) and anti-mouse IgG (1:25,000) secondary antibodies for 1 h at room temperature and detected using an advanced electrochemiluminescence western blot detection kit (Amersham, Uppsala, Sweden). The intensities of the protein bands were measured using ImageJ software (http://rsb.info.nih.gov/ij/, NIH, MD, USA). All experiments were performed in two separate replicates.

### 2.12. ELISA Analysis

To analyze the activities of IL-6 in MSCs following VCA treatment, PD-MSCs and BM-MSCs were seeded in 6-well culture plates (BD Biosciences) at 1.0 × 10^4^ cells per well. After 24 h, the cells were treated with various concentrations of VCA (0, 1, 5, 10 and 100 pg/mL) for 48 h. The culture media were collected and preserved at −80 °C, and the activities of IL-6 were measured using human-specific ELISA kits (BD Biosciences) according to the manufacturer’s instructions. All experiments were performed in two separate replicates.

### 2.13. Immunofluorescence

PD-MSCs (1.0 × 10^4^) were seeded on cover slides in 6-well plates with culture medium for 24 h and treated with different concentrations of VCA (0, 1, 5, 10 pg/mL) and neutralizing anti-IL-6 for 48 h. The cover slides were fixed in 100% methanol for 10 min, incubated with blocking solution (Dako, Carpinteria, CA, USA) for 30 min and incubated overnight at 4 °C with anti-ki67 (Dako). The next day, the cover slides were incubated at room temperature with an Alexa 594-conjugated antibody (Invitrogen) for 1 h, followed by staining with 1 µg/mL PI (Vector Laboratories, Burlingame, CA, USA). Images were analyzed using an microscope (AMG, Seattle, WA, USA). The number of positive cells in at least seven randomly nonoverlapping fields on the slides was counted at a magnification of x100.

### 2.14. Statistical Analysis

Statistical analyses were performed with Student’s *t*-test or ANOVA using R. *p* value < 0.05 was considered statistically significant.

## 3. Results

### 3.1. Characterization of PD-MSCs Isolated from Normal Term Placenta

**Figure 1:**
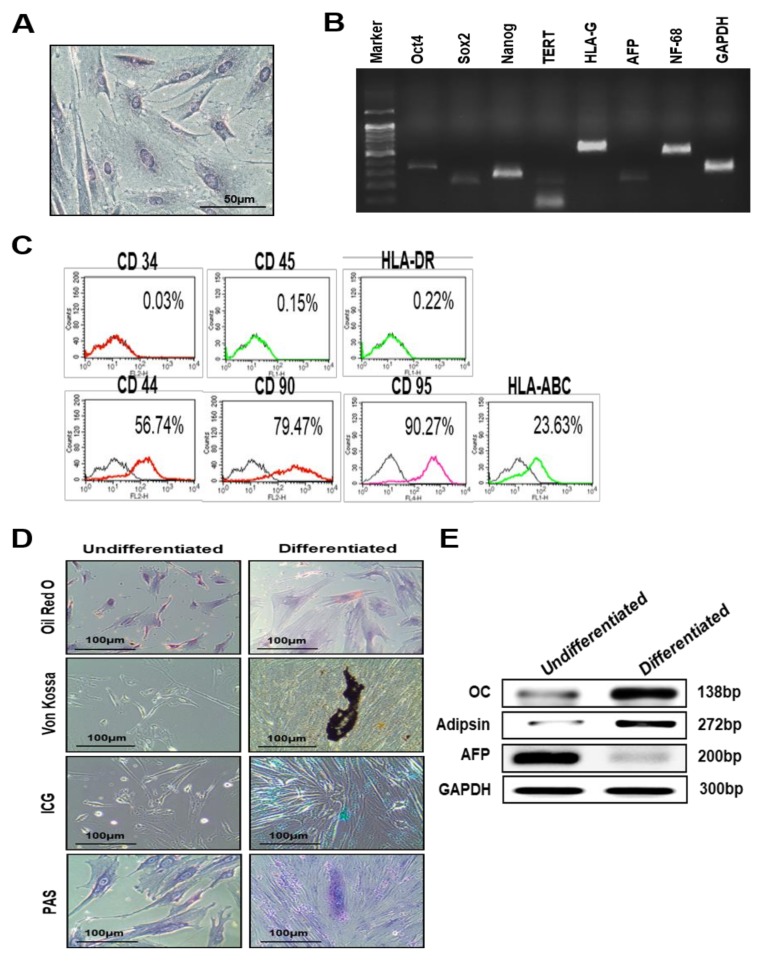
Characterization of PD-MSCs isolated from normal human term placenta. (**A**) Morphology of PD-MSCs from normal term placenta. (**B**) Expression of stem cell markers in PD-MSCs measured by RT-PCR. (**C**) Immunophenotyping of PD-MSCs by flow cytometry. Representative histograms for CD markers are shown (PE, red line; FITC, green line; and APC, pink line). The respective isotype control is shown as a black line. (**D**) The differentiation potentials of PD-MSCs were verified by a functional assay after culturing for 3 weeks (Oil Red O staining for adipocyte differentiation (magnification, ×200), von Kossa staining for osteocyte differentiation, ICG uptake and PAS staining for hepatocyte differentiation) (magnification, ×100). Scale bars: 100 µm. (**E**) mRNA expression of differentiation markers in PD-MSCs after culturing for 3 weeks was confirmed by RT-PCR. GAPDH was used as an internal control. Arrowheads indicate a positive reaction for each staining.

First, we characterized PD-MSCs isolated from normal term placentas. The morphology of the PD-MSCs was similar to that of other MSCs, such as BM-MSCs (spindle shape and fibroblast-like morphology) (Figure 1A). Next, we evaluated mRNA levels and immunophenotypes using RT-PCR and FACS analysis. mRNA expression of the stemness markers Oct, Nanog, and Sox2, the germ lineage-specific markers NF68, Cardiac, and AFP and the immune-specific marker HLA-G was detected in PD-MSCs (Figure 1B). Additionally, immunophenotype analysis revealed that surface antigens associated with mesenchymal stem cells, such as CD-44, CD-90, CD-95 and HLA-ABC, were expressed by these PD-MSCs (Figure 1C). Thus, we confirmed the differentiation potential of PD-MSCs into multiple lineages. After 21 days in specific induction medium, the morphology of adipogenically differentiated cells was transformed into a round shape with lipid-containing vacuoles, as visualized using Oil Red O (Figure 1D). Calcium deposits, indicative of osteocytes, were significantly accumulated in osteogenic differentiated cells, as revealed by von Kossa staining (Figure 1D). Differentiation of the PD-MSCs into hepatocytes was also evaluated by ICG uptake and PAS staining. ICG uptake was significantly increased in hepatogenic-differentiated cells compared with undifferentiated cells. Additionally, glycogen stores were increased in hepatogenic-differentiated cells compared with undifferentiated cells (Figure 1D). Moreover, the mRNA expression levels of specific differentiation markers of adipocytes, (adipsin), osteocytes (osteocalcin; OC) and hepatocytes (albumin and cyp3A4) were increased in differentiated cells compared with undifferentiated cells, and mRNA expression of the early hepatocyte marker AFP was decreased in differentiated cells compared with undifferentiated cells (Figure 1E). These findings suggest that PD-MSCs isolated from normal term placenta are an alternative cell source because their characteristics are similar to those of other MSCs.

### 3.2. Effect of VCA on PD-MSC Self-Renewal

**Figure 2:**
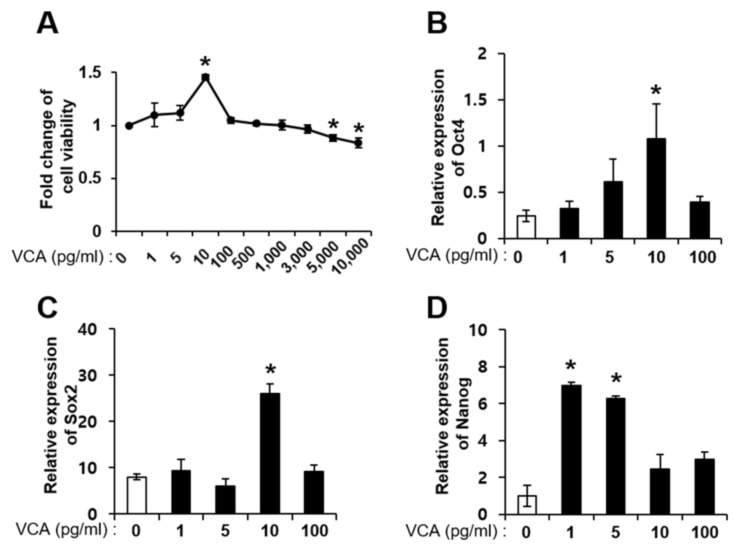
Effect of VCA on the viability and self-renewal of PD-MSCs. (**A**) Proliferation assay in PD-MSCs at the concentration of VCA determined by MTT analysis. Expression of (**B**) Oct4, (**C**) Sox2 and (**D**) Nanog in PD-MSCs treated with VCA, as determined by western blotting. All reactions were performed in triplicate. Data are shown as the mean ± standard error (S.E.); * indicates a significant difference compared to the untreated group (*p* < 0.05).

In general, the pharmacokinetics of natural products show a biphasic effect, depending on the cell type or conditions. Therefore, we performed an MTT assay on PD-MSCs treated with several concentrations of VCA (0–10,000 pg/mL) to confirm the effect of VCA on proliferation. The viability of PD-MSCs was significantly enhanced up to 1.5-fold in the 10 pg/mL VCA-treated group compared to the untreated group (*p* < 0.05); moreover, cell viability of PD-MSCs was significantly decreased in the groups treated with 5000 and 10,000 pg/mL VCA compared with the no treatment group (Figure 2A). Similar to PD-MSCs, the viability of BM-MSCS was significantly enhanced at a low concentration of VCA (5 pg/mL) and decreased at 10,000 pg/mL VCA compared with no treatment (*p* < 0.05) (Supplementary Figure S1A). Next, we analyzed expression of stemness-related markers such as Oct4, Sox2 and Nanog in PD-MSCs treated with VCA because their viabilities were increased at low concentrations. Oct4 and Sox2 mRNA expression was significantly increased in PD-MSCs treated with VCA (10 pg/mL) up to 4.4-and 2.7-fold compared with untreated PD-MSCs (*p* < 0.05) (Figure 2B,C). Nanog mRNA expression was also significantly increased in VCA (1 and 5 pg/mL)-treated PD-MSCs (*p* < 0.05) (Figure 2D). Furthermore, the expression levels of Oct4, Sox2, and Nanog were similar in BM-MSCs and PD-MSCs (Supplementary Figure S1B–D). These findings suggest that a low concentration of VCA increases the proliferation of MSCs but that a high concentration of VCA decreases it. The increased proliferation of MSCs at the low VCA concentration appears to be affected by upregulation of stemness-related markers, such as Oct4, Sox2 and Nanog.

### 3.3. VCA Enhances the Self-Renewal of PD-MSCs by IL-6 Production

**Figure 3:**
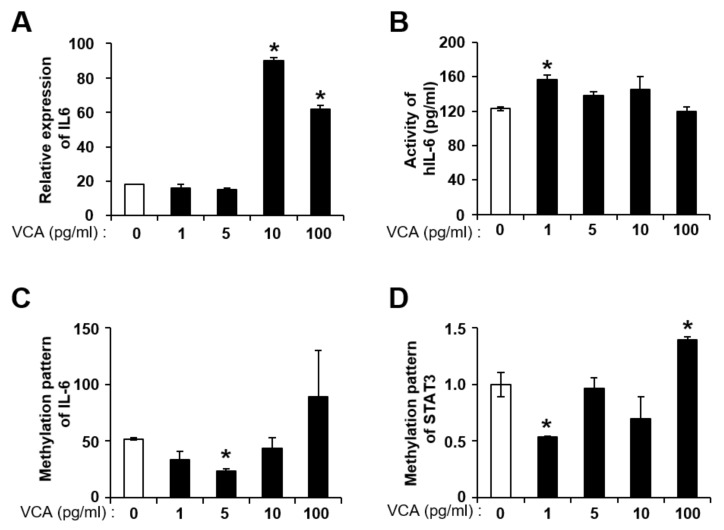
Effect of VCA on alteration of the IL-6/STAT3 signaling pathway in PD-MSCs. (**A**) mRNA and (**B**) protein expression of human IL-6 in PD-MSCs treated with VCA, as determined by qRT-PCR and ELISA, respectively. 18S rRNA was used as an internal control. (**C**) IL-6 and (**D**) STAT3 were assessed by qMSP. Relative quantification of methylation is expressed as the percentage of the methylation ratio (PMR). All reactions were performed in triplicate. Data are shown as the mean ± S.E.; * indicates a significant difference compared to the untreated group (*p* < 0.05).

In previous studies, we demonstrated that VCA regulates the self-renewal of PD-MSCs via autophagic mechanisms [25]. Therefore, we analyzed whether the expression and activity of IL-6 in PD-MSCs were altered by VCA treatment. mRNA expression of IL-6 was significantly increased up to 3~4-fold in PD-MSCs treated with VCA (10 and 100 pg/mL) compared with untreated PD-MSCs (*p* < 0.05) (Figure 3A). IL-6 activity in PD-MSCs was significantly increased with 1 pg/mL VCA compared with no treatment (*p* < 0.05) (Figure 3B). In the case of BM-MSCs, expression of IL-6 was significantly increased by 1.2-fold in the 5 pg/mL VCA-treated group compared with the untreated group (*p* < 0.05) (Supplementary Figure S2A), but there was no significant increase in the activity of IL-6 at all VCA concentrations (Supplementary Figure S2B). 

Because gene expression is regulated by genetic and epigenetic factors, expression patterns are structurally and functionally heterogeneous because of the surrounding environment [47]. Recently, Nardelli C et al. showed that epigenetic changes in amniotic mesenchymal stem cells under obesity conditions regulate the altered expression of several pathways, such as ILs, the cell cycle and telomere maintenance [48]. Therefore, we analyzed the methylation pattern of IL-6/STAT3 genes using qMSP to confirm the expression patterns of IL-6/STAT3 in PD-MSCs treated with VCA. IL-6 was demethylated with 10 pg/mL VCA treatment; however, the DNA methylation status of IL-6 was significantly increased in PD-MSCs treated with 100 pg/mL VCA (*p* < 0.05) (Figure 3C). In addition, the STAT3 gene was significantly demethylated in PD-MSCs treated with 1 pg/mL VCA (*p* < 0.05) (Figure 3D). Although the IL-6 gene was demethylated in BM-MSCs with 5 pg/mL VCA, similar to PD-MSCs, methylation of STAT3 in BM-MSCs exposed to VCA treatment was lower than that of the control (*p* < 0.05) (Supplementary Figure S2D). These results indicate that VCA may enhance the proliferation of MSCs through epigenetic regulation of IL-6/STAT3.

### 3.4. Inhibition of IL-6 Decreases the Proliferation of PD-MSCs Stimulated with VCA by Regulating Stemness Markers

**Figure 4:**
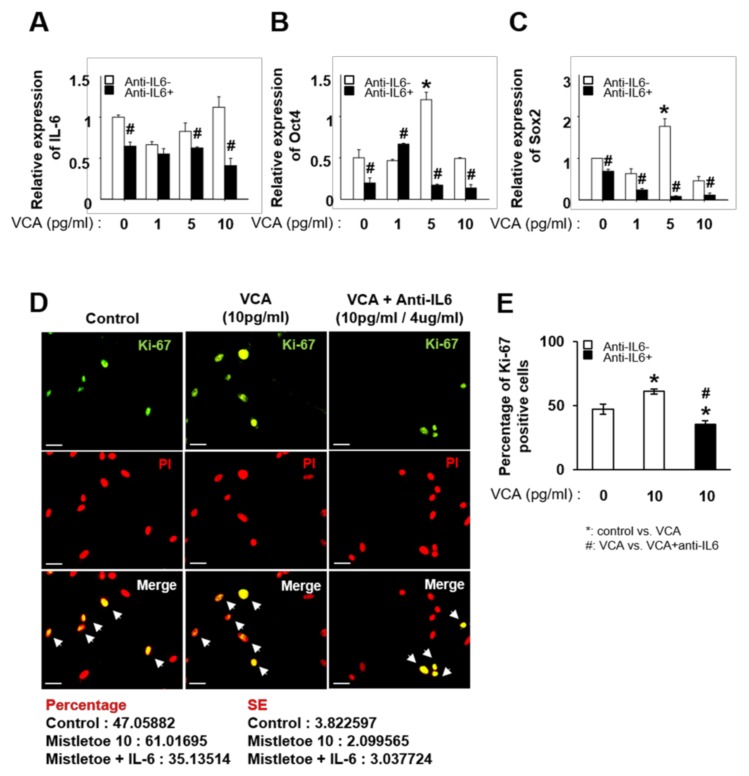
Effect of IL-6 on the self-renewal activity of PD-MSCs treated with VCA. Relative mRNA expression of (**A**) IL-6, (**B**) Oct4 and (**C**) Sox2 in PD-MSCs treated with VCA and anti-IL-6 (4 µg/mL), as determined by qRT-PCR. (**D**) Localization and (**E**) quantification of Ki-67+ PD-MSCs treated with only VCA or a combination of VCA and anti-IL-6, as determined by immunofluorescence. Scale bar = 50 µm. Green represents Ki67-positive nuclei in PD-MSCs. PI was used as the counterstain. Ki-67 + PD-MSCs were counted using ImageJ. All experiments were performed in triplicate. Data are shown as the mean ± S.E.; * indicates a significant difference compared to the untreated group; # indicates a significant difference compared to the corresponding VCA-treated group without anti-IL-6 treatment.

We confirmed that VCA enhances the self-renewal activity of PD-MSCs via increased expression of IL-6. Next, we demonstrated whether the increased self-renewal activity of PD-MSCs exposed to VCA treatment is suppressed by IL-6 neutralization using an anti-IL-6 antibody. As shown in Figure 4A, expression of IL-6 in PD-MSCs was significantly decreased by neutralization of IL-6, regardless of VCA treatment (*p* < 0.05, Figure 4A). Overall, expression of Oct4 and Sox2 in PD-MSCs cultured with VCA was significantly decreased by neutralization of IL-6 compared to the control condition (*p* < 0.05) (Figure 4B,C). Based on the results, the self-renewal ability of PD-MSCs is suppressed by neutralization of IL-6. Interestingly, Ki-67, which is a representative marker for proliferation, was expressed in the nucleus of PD-MSCs treated with 10 pg/mL VCA, and expression was decreased by IL-6 neutralization (Figure 4D,E). These results indicate that IL-6 plays a role in the proliferation of PD-MSCs through the expression of stemness markers such as Oct4 and Sox2.

### 3.5. VCA Enhances the Self-Renewal of PD-MSCs through IL-6-Mediated STAT3 Signaling

**Figure 5:**
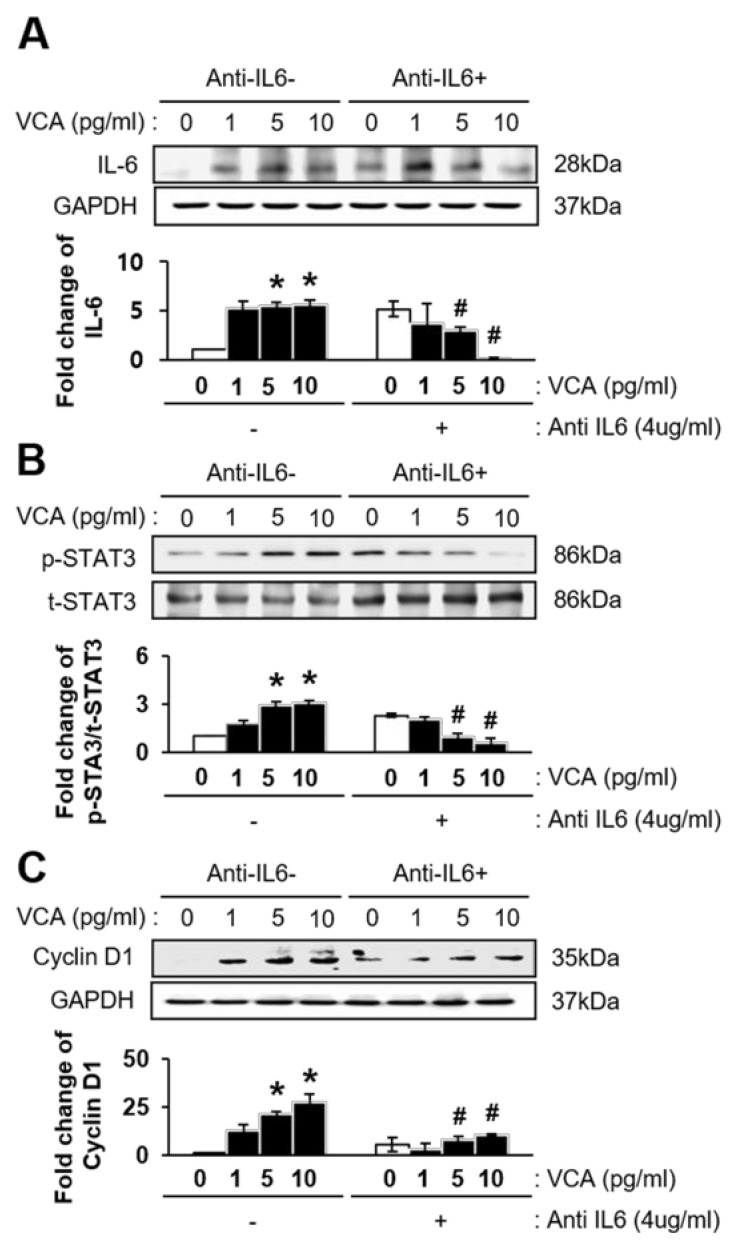
Effect of IL-6/STAT3 on the cell cycle of PD-MSCs treated with VCA. Expression of (**A**) IL-6, (**B**) phosphorylated or total STAT3 and (**C**) Cyclin D1 in PD-MSCs treated with VCA and anti-IL-6, as determined by western blotting. Band intensity was quantified based on the internal control, GAPDH. * indicates a significant difference compared to the untreated group; # indicates a significant difference compared to the corresponding VCA-treated group without anti-IL-6 treatment.

To confirm the effect of IL-6-mediated STAT3 signaling on the self-renewal of PD-MSCs treated with VCA, we first examined expression of IL-6/STAT3 in PD-MSCs treated with both VCA and the anti-IL-6 antibody. As shown in Figure 5A, expression of IL-6 in PD-MSCs exposed to VCA was increased, whereas neutralization of IL-6 with the anti-IL-6 antibody (4 µg/mL) suppressed expression of IL-6, regardless of VCA treatment (*p* < 0.05). Furthermore, the ratio of phosphorylated STAT3 (p-STAT3) to total STAT3 (t-STAT3) in PD-MSCs was gradually increased by VCA treatment (*p* < 0.05), though the ratio was significantly decreased by neutralization of IL-6 with the anti-IL-6 antibody (*p* < 0.05) (Figure 5B). Next, we examined whether IL-6 neutralization affects the proliferation of PD-MSCs exposed to VCA. Expression of Cyclin D1, which is related to the G1/S-phase transition of the cell cycle, was significantly increased by VCA treatment but dramatically suppressed by anti-IL-6 antibody treatment (*p* < 0.05) (Figure 5C). These data indicate that a low concentration of VCA (1–10 pg/mL) enhances the self-renewal ability of MSCs through IL-6/STAT3 signaling.

## 4. Discussion

To our knowledge, we are the first to demonstrate that low concentrations of VCA directly regulate methylation of the genes encoding the proinflammatory cytokines IL-6 and STAT3. VCA is composed of two independent subunits, the A-chain and B-chain, which exert cytotoxic effects by regulating cell death in cancer cells [18]. However, a nontoxic concentration of VCA stimulates proinflammatory cytokines such as IL-1α, β, IL-6, and IL-8 and regulates the proliferation of murine splenocytes and human peripheral blood mononuclear cells [49,50,51]. Nevertheless, there are few studies on the effects of VCA on the proliferation of MSCs. In previous reports, we demonstrated that a low concentration of VCA increased the proliferative activity of PD-MSCs by regulating the Nanog-dependent cell cycle through autophagic mechanisms but that VCA, regardless of the concentration, decreased the proliferative activity of cancer cells [25]. In general, the mechanism by which VCA affects MSC proliferation and epigenetic regulation of gene expression should be clarified.

The interleukin family is well known to activate target genes involved in proliferation, differentiation, and apoptosis. Recently, it was reported that IL-1β regulates cell proliferation through stemness markers such as Bmi-1, Lgr-5, c-myc and Nanog in cancer stem cells [52,53]. Additionally, Li et al. demonstrated that low concentrations of proinflammatory cytokines, including IL-6 and IL-8, can stimulate the proliferation of PD-MSCs in a dose-dependent manner, whereas anti-inflammatory cytokines, including IL-4 and HGF, inhibit the proliferation of PD-MSCs. However, these results did not reveal the relationship between proinflammatory and anti-inflammatory cytokines. [54]. Our findings are in agreement with these results: methylation of the gene encoding the proinflammatory cytokine IL-6 induced by low concentrations of VCA can promote the proliferation of MSCs by regulating expression of downstream genes.

Methylation patterns modulate gene expression by altering CpG regions in the promoter of a target gene, promoting or suppressing several processes, such as proliferation, adhesion motility and signal transduction [55]. In particular, DNA methylation is a key regulator that silences expression of interleukin genes, including IL-1b, IL-6 and IL-8 [56,57,58]. In a previous study, we demonstrated that PD-MSCs mediate the hypomethylation of IL-6 and IL-6R, but not STAT3, promoting the proliferation of hepatocytes [59]. In this study, we showed that the demethylation of IL-6 and STAT3 induced by low concentrations of VCA increased the proliferation of MSCs via IL-6/STAT3 signaling. Furthermore, neutralization of IL-6 under low concentrations of VCA reduced MSC proliferation and STAT3 phosphorylation.

Activation of STAT3 induced by LIF, which is an IL-6 class cytokine, is a critical factor for the self-renewal ability of ESCs that is achieved through the balance between differentiation and self-renewal [60]. Previous reports suggest that the demethylation of STAT3 may be associated with the methylation of various genes, including IL-6. Increased IL-6 expression by changed methylation patterns promotes cell proliferation in lung cancer cells by regulating the methylation of p53 and p21 genes through DNA methyltransferase1 (DNMT1), though expression of CDKs and CyclinD1 remains unchanged [61]. Although VCA increased expression of Cyclin D1 and IL-6/STAT3 pathway components and expression of Cyclin D1 was reduced by neutralization of IL-6, a slight difference in Cyclin D1 expression was found between BM-MSCs and PD-MSCs in the present study. These results indicate that expression of Cyclin D1 in PD-MSCs is indirectly controlled by IL-6 but that its expression in BM-MSCs is directly regulated by IL-6 and STAT3, similar to the finding of Won et al. [62]. Based on these data, we suggest that a better understanding of the mechanism by which IL-6/STAT3/Cyclin D1 and DNMT1 are involved in the cell cycle and DNA methylation of MSCs is necessary.

## 5. Conclusions

In summary, the present study shows that PD-MSCs might be a useful screening tool for pharmacologically interesting compounds. Additionally, the findings suggest that a low concentration of VCA promotes the self-renewal of PD-MSCs through stemness markers by upregulating Oct4, Sox2 and Nanog. Additionally, VCA can stimulate expression of Cyclin D1 by regulating the IL-6/STAT3 pathway via methylation. Therefore, the data improve our understanding of the self-renewal mechanism of MSCs via epigenetic regulation. Additionally, VCA enhanced the self-renewal ability of MSCs, such as PD-MSCs and BM-MSCs, and may be a useful natural supplement to regulate the environment of cells for screening studies of natural product active ingredients.

## Supplementary Materials

The following are available online at https://www.mdpi.com/2072-6643/11/11/2604/s1, Table S1: RT-PCR primers used in the study; Figure S1: Effect of VCA on the viability and self-renewal of BM-MSCs; Figure S2: Effect of VCA on the alteration of the IL-6/STAT3 signaling pathway in BM-MSCs.

## References

[1] Pittenger M.F., Mackay A.M., Beck S.C., Jaiswal R.K., Douglas R., Mosca J.D., Moorman M.A., Simonetti D.W., Craig S., Marshak D.R. (1999). Multilineage potential of adult human mesenchymal stem cells. Science.

[2] Kaplan J.M., Youd M.E., Lodie T.A. (2011). Immunomodulatory activity of mesenchymal stem cells. Curr. Stem Cell Res. Ther..

[3] De Miguel M.P., Fuentes-Julian S., Blazquez-Martinez A., Pascual C.Y., Aller M.A., Arias J., Arnalich-Montiel F. (2012). Immunosuppressive properties of mesenchymal stem cells: Advances and applications. Curr. Mol. Med..

[4] Karp J.M., Leng Teo G.S. (2009). Mesenchymal stem cell homing: The devil is in the details. Cell Stem Cell.

[5] Selmani Z., Naji A., Zidi I., Favier B., Gaiffe E., Obert L., Borg C., Saas P., Tiberghien P., Rouas-Freiss N. (2008). Human leukocyte antigen-G5 secretion by human mesenchymal stem cells is required to suppress T lymphocyte and natural killer function and to induce CD4+CD25highFOXP3+ regulatory T cells. Stem Cells.

[6] Luan X., Li G., Wang G., Wang F., Lin Y. (2013). Human placenta-derived mesenchymal stem cells suppress T cell proliferation and support the culture expansion of cord blood CD34(+) cells: A comparison with human bone marrow-derived mesenchymal stem cells. Tissue Cell.

[7] Lee J.M., Jung J., Lee H.J., Jeong S.J., Cho K.J., Hwang S.G., Kim G.J. (2012). Comparison of immunomodulatory effects of placenta mesenchymal stem cells with bone marrow and adipose mesenchymal stem cells. Int. Immunopharmacol..

[8] Fazekasova H., Lechler R., Langford K., Lombardi G. (2011). Placenta-derived MSCs are partially immunogenic and less immunomodulatory than bone marrow-derived MSCs. J. Tissue Eng. Regen. Med..

[9] Pisciotta A., Riccio M., Carnevale G., Beretti F., Gibellini L., Maraldi T., Cavallini G.M., Ferrari A., Bruzzesi G., De Pol A. (2012). Human serum promotes osteogenic differentiation of human dental pulp stem cells in vitro and in vivo. PLoS ONE.

[10] Hong L., Zhang G., Sultana H., Yu Y., Wei Z. (2011). The effects of 17-beta estradiol on enhancing proliferation of human bone marrow mesenchymal stromal cells in vitro. Stem Cells Dev..

[11] Gharibi B., Hughes F.J. (2012). Effects of medium supplements on proliferation, differentiation potential, and in vitro expansion of mesenchymal stem cells. Stem Cells Transl. Med..

[12] Chieregato K., Castegnaro S., Madeo D., Astori G., Pegoraro M., Rodeghiero F. (2011). Epidermal growth factor, basic fibroblast growth factor and platelet-derived growth factor-bb can substitute for fetal bovine serum and compete with human platelet-rich plasma in the ex vivo expansion of mesenchymal stromal cells derived from adipose tissue. Cytotherapy.

[13] He X., H’Ng S.C., Leong D.T., Hutmacher D.W., Melendez A.J. (2010). Sphingosine-1-phosphate mediates proliferation maintaining the multipotency of human adult bone marrow and adipose tissue-derived stem cells. J. Mol. Cell Biol.

[14] Zeng H.P., Wang T.T., Chen W., Wang C.Y., Chen D.F., Shen J.G. (2008). Characterization of chemical components in extracts from Si-wu decoction with proliferation-promoting effects on rat mesenchymal stem cells. Bioorgan. Med. Chem..

[15] Zhou J.H., Zeng H.P., Chen D.F., Li H., Li X.C., Li Y.W., Du S.H. (2006). Effect of extract components from Plastrum testudinis and their combination component on proliferation of rat mesenchymal stem cell in vitro. Zhong Yao Cai.

[16] Li X., Zhou J., Li H., Du S., Li Y., Huang L., Chen D. (2005). Study on proliferation effect of extracts of Piper longum on mesenchymal stem cells of rat bone marrow and the relationship to chemical functional groups. Zhong Yao Cai.

[17] Potu B.K., Bhat K.M., Rao M.S., Nampurath G.K., Chamallamudi M.R., Nayak S.R., Muttigi M.S. (2009). Petroleum ether extract of Cissus quadrangularis (Linn.) enhances bone marrow mesenchymal stem cell proliferation and facilitates osteoblastogenesis. Clinics.

[18] Kim Y., Kim I., Park C.H., Kim J.B. (2018). Korean mistletoe lectin enhances natural killer cell cytotoxicity via upregulation of perforin expression. Asian Pac. J. Allergy Immunol..

[19] Han S.Y., Hong C.E., Kim H.G., Lyu S.Y. (2015). Anti-cancer effects of enteric-coated polymers containing mistletoe lectin in murine melanoma cells in vitro and in vivo. Mol. Cell. Biochem..

[20] Khil L.Y., Kim W., Lyu S., Park W.B., Yoon J.W., Jun H.S. (2007). Mechanisms involved in Korean mistletoe lectin-induced apoptosis of cancer cells. World J. Gastroenterol..

[21] Kim B.K., Choi M.J., Park K.Y., Cho E.J. (2010). Protective effects of Korean mistletoe lectin on radical-induced oxidative stress. Biol. Pharm. Bull..

[22] Park H.J., Hong J.H., Kwon H.J., Kim Y., Lee K.H., Kim J.B., Song S.K. (2010). TLR4-mediated activation of mouse macrophages by Korean mistletoe lectin-C (KML-C). Biochem. Biophys. Res. Commun..

[23] Lee C.H., Kim J.K., Kim H.Y., Park S.M., Lee S.M. (2009). Immunomodulating effects of Korean mistletoe lectin in vitro and in vivo. Int. Immunopharmacol..

[24] Lyu S.Y., Choi J.H., Lee H.J., Park W.B., Kim G.J. (2013). Korean mistletoe lectin promotes proliferation and invasion of trophoblast cells through regulation of Akt signaling. Reprod. Toxicol..

[25] Choi J.H., Lyu S.Y., Lee H.J., Jung J., Park W.B., Kim G.J. (2012). Korean mistletoe lectin regulates self-renewal of placenta-derived mesenchymal stem cells via autophagic mechanisms. Cell Prolif..

[26] Zon L.I. (2008). Intrinsic and extrinsic control of haematopoietic stem-cell self-renewal. Nature.

[27] Wilson A., Murphy M.J., Oskarsson T., Kaloulis K., Bettess M.D., Oser G.M., Pasche A.C., Knabenhans C., Macdonald H.R., Trumpp A. (2004). c-Myc controls the balance between hematopoietic stem cell self-renewal and differentiation. Genes Dev..

[28] Boiani M., Scholer H.R. (2005). Regulatory networks in embryo-derived pluripotent stem cells. Nat. Rev. Mol. Cell Biol..

[29] Boyer L.A., Lee T.I., Cole M.F., Johnstone S.E., Levine S.S., Zucker J.P., Guenther M.G., Kumar R.M., Murray H.L., Jenner R.G. (2005). Core transcriptional regulatory circuitry in human embryonic stem cells. Cell.

[30] Fan Y.X., Gu C.H., Zhang Y.L., Zhong B.S., Wang L.Z., Zhou Z.R., Wang Z.Y., Jia R.X., Wang F. (2013). Oct4 and Sox2 overexpression improves the proliferation and differentiation of bone mesenchymal stem cells in Xiaomeishan porcine. Genet. Mol. Res..

[31] Lee J., Go Y., Kang I., Han Y.M., Kim J. (2010). Oct-4 controls cell-cycle progression of embryonic stem cells. Biochem. J..

[32] Coronado D., Godet M., Bourillot P.Y., Tapponnier Y., Bernat A., Petit M., Afanassieff M., Markossian S., Malashicheva A., Iacone R. (2013). A short G1 phase is an intrinsic determinant of naive embryonic stem cell pluripotency. Stem Cell Res..

[33] Johnson D.E., O’Keefe R.A., Grandis J.R. (2018). Targeting the IL-6/JAK/STAT3 signalling axis in cancer. Nat. Rev. Clin. Oncol..

[34] Chen P., Huang L., Zhang Y., Qiao M., Yuan Y. (2010). SiRNA-mediated PIAS1 silencing promotes inflammatory response and leads to injury of cerulein-stimulated pancreatic acinar cells via regulation of the P38MAPK signaling pathway. Int. J. Mol. Med..

[35] Wehbe H., Henson R., Meng F., Mize-Berge J., Patel T. (2006). Interleukin-6 contributes to growth in cholangiocarcinoma cells by aberrant promoter methylation and gene expression. Cancer Res..

[36] Zhang X., Zeng Y., Qu Q., Zhu J., Liu Z., Ning W., Zeng H., Zhang N., Du W., Chen C. (2017). PD-L1 induced by IFN-gamma from tumor-associated macrophages via the JAK/STAT3 and PI3K/AKT signaling pathways promoted progression of lung cancer. Int. J. Clin. Oncol..

[37] Gao S.P., Mark K.G., Leslie K., Pao W., Motoi N., Gerald W.L., Travis W.D., Bornmann W., Veach D., Clarkson B. (2007). Mutations in the EGFR kinase domain mediate STAT3 activation via IL-6 production in human lung adenocarcinomas. J. Clin. Investig..

[38] Yeh H.H., Lai W.W., Chen H.H., Liu H.S., Su W.C. (2006). Autocrine IL-6-induced Stat3 activation contributes to the pathogenesis of lung adenocarcinoma and malignant pleural effusion. Oncogene.

[39] Meng F., Yamagiwa Y., Ueno Y., Patel T. (2006). Over-expression of interleukin-6 enhances cell survival and transformed cell growth in human malignant cholangiocytes. J. Hepatol..

[40] Okada K., Shimizu Y., Nambu S., Higuchi K., Watanabe A. (1994). Interleukin-6 functions as an autocrine growth factor in a cholangiocarcinoma cell line. J. Gastroenterol. Hepatol..

[41] Dandrea M., Donadelli M., Costanzo C., Scarpa A., Palmieri M. (2009). MeCP2/H3meK9 are involved in IL-6 gene silencing in pancreatic adenocarcinoma cell lines. Nucleic Acids Res..

[42] Armenante F., Merola M., Furia A., Palmieri M. (1999). Repression of the IL-6 gene is associated with hypermethylation. Biochem. Biophys. Res. Commun..

[43] Choi B., Chun E., Kim S.Y., Kim M., Lee K.Y., Kim S.J. (2012). Notch-induced hIL-6 production facilitates the maintenance of self-renewal of hCD34+ cord blood cells through the activation of Jak-PI3K-STAT3 pathway. Am. J. Pathol..

[44] Kovacs E. (2010). Investigation of the proliferation, apoptosis/necrosis, and cell cycle phases in several human multiple myeloma cell lines. Comparison of Viscum album QuFrF extract with vincristine in an in vitro model. Sci. World J..

[45] Lee M.J., Jung J., Na K.H., Moon J.S., Lee H.J., Kim J.H., Kim G.I., Kwon S.W., Hwang S.G., Kim G.J. (2010). Anti-fibrotic effect of chorionic plate-derived mesenchymal stem cells isolated from human placenta in a rat model of CCl(4)-injured liver: Potential application to the treatment of hepatic diseases. J. Cell Biochem..

[46] Lyu S.Y., Park S.M., Choung B.Y., Park W.B. (2000). Comparative study of Korean (Viscum album var. coloratum) and European mistletoes (Viscum album). Arch. Pharm. Res..

[47] Jaenisch R., Bird A. (2003). Epigenetic regulation of gene expression: How the genome integrates intrinsic and environmental signals. Nat. Genet..

[48] Nardelli C., Granata I., Iaffaldano L., D’Argenio V., Del Monaco V., Maruotti G.M., Omodei D., Del Vecchio L., Martinelli P., Salvatore F. (2017). miR-138/miR-222 Overexpression Characterizes the miRNome of Amniotic Mesenchymal Stem Cells in Obesity. Stem Cells Dev..

[49] Lyu S.Y., Park W.B. (2007). Effects of Korean mistletoe lectin (Viscum album coloratum) on proliferation and cytokine expression in human peripheral blood mononuclear cells and T-lymphocytes. Arch. Pharm. Res..

[50] Lyu S.Y., Park W.B. (2006). Mistletoe lectin (Viscum album coloratum) modulates proliferation and cytokine expressions in murine splenocytes. J. Biochem. Mol. Biol..

[51] Hajto T., Hostanska K., Frei K., Rordorf C., Gabius H.J. (1990). Increased secretion of tumor necrosis factors alpha, interleukin 1, and interleukin 6 by human mononuclear cells exposed to beta-galactoside-specific lectin from clinically applied mistletoe extract. Cancer Res..

[52] Li Y., Wang L., Pappan L., Galliher-Beckley A., Shi J. (2012). IL-1beta promotes stemness and invasiveness of colon cancer cells through Zeb1 activation. Mol. Cancer.

[53] Wang L., Liu Z., Li Y., Pappan L., Galliher-Beckley A., Shi J. (2012). Pro-inflammatory cytokine interleukin-1beta promotes the development of intestinal stem cells. Inflamm. Res..

[54] Li D., Wang G.Y., Dong B.H., Zhang Y.C., Wang Y.X., Sun B.C. (2007). Biological characteristics of human placental mesenchymal stem cells and their proliferative response to various cytokines. Cells Tissues Organs.

[55] Pogribny I.P., Beland F.A. (2013). DNA methylome alterations in chemical carcinogenesis. Cancer Lett..

[56] Poplutz M.K., Wessels I., Rink L., Uciechowski P. (2014). Regulation of the Interleukin-6 gene expression during monocytic differentiation of HL-60 cells by chromatin remodeling and methylation. Immunobiology.

[57] Tekpli X., Landvik N.E., Anmarkud K.H., Skaug V., Haugen A., Zienolddiny S. (2013). DNA methylation at promoter regions of interleukin 1B, interleukin 6, and interleukin 8 in non-small cell lung cancer. Cancer Immunol. Immunother..

[58] Nile C.J., Read R.C., Akil M., Duff G.W., Wilson A.G. (2008). Methylation status of a single CpG site in the IL6 promoter is related to IL6 messenger RNA levels and rheumatoid arthritis. Arthritis Rheum..

[59] Jung J., Moon J.W., Choi J.H., Lee Y.W., Park S.H., Kim G.J. (2015). Epigenetic Alterations of IL-6/STAT3 Signaling by Placental Stem Cells Promote Hepatic Regeneration in a Rat Model with CCl4-induced Liver Injury. Int. J. Stem Cells.

[60] Burdon T., Smith A., Savatier P. (2002). Signalling, cell cycle and pluripotency in embryonic stem cells. Trends Cell Biol..

[61] Liu C.C., Lin J.H., Hsu T.W., Su K., Li A.F., Hsu H.S., Hung S.C. (2015). IL-6 enriched lung cancer stem-like cell population by inhibition of cell cycle regulators via DNMT1 upregulation. Int. J. Cancer.

[62] Won C., Lee C.S., Lee J.K., Kim T.J., Lee K.H., Yang Y.M., Kim Y.N., Ye S.K., Chung M.H. (2010). CADPE suppresses cyclin D1 expression in hepatocellular carcinoma by blocking IL-6-induced STAT3 activation. Anticancer Res..

