# Supplementary material for: Alterations in IL-6/STAT3 Signaling by Korean Mistletoe Lectin Regulate the Self-Renewal Activity of Placenta-Derived Mesenchymal Stem Cells
Source: Nutrients. 2019 Oct 30;11(11):2604. doi: 10.3390/nu11112604 (PMC6893712; doi:10.3390/nu11112604)
Supplement: Supplementary file 1 [file nutrients-11-02604-s001.pdf]

Table S1. RT-PCR primers used in the study.

Gene	Primer sequences (5'-3')	Primer (bp)	Tm (°C)
Oct4	F: 5'-AGTGAGAGGCAACCTGGAGA-3'<br>R: 5'-GTGAAGTGAGGGCTCCCATA-3'	273	54
Nanog	F: 5'-TTCTTGACTGGGACCTTGTC-3'<br>R: 5'-GCTTGCCTTGCTTTGAAGCA-3'	300	54
Sox2	F: 5'-AGAACCCCAAGATGCACAAC-3'<br>R: 5'-GGGCAGCGTGTACTTATCCT-3'	200	52
NF-68	F: 5'-GAGTGAAATGGCACGATACCTA-3'<br>R: 5'-TTTCCTCTCCTTCTTCTTCACCTTC-3'	700	58
AFP	F: 5'-ATGCTGCAAACTGACCACGC-3'<br>R: 5'-GCTTCGCTTTGCCAATGCTT-3'	500	55
HLA-G	F: 5'-GCGGCTACTACAACCAGAGC-3'<br>R: 5'-GCACATGGCACGTGTATCTC-3'	900	58
TERT	F: 5'-GAGCTGACGTGGAAGATGAG-3'<br>R: 5'-CTTCAAGTGCTGTCTGATTCCAATG-3'	300	55
OC	F: 5'-GCAGCGAGGTAGTGAAGAGA-3'<br>R: 5'-CGATGTGGTCAGCCAACT-3'	138	58
Adipsin	F: 5'-GGTCACCCAAGCAACAAAGT-3'<br>R: 5'-CCTCCTGCGTTCAAGTCATC-3'	272	59
GAPDH	F: 5'-CGAGATCCCTCCAAAATCAA-3'<br>R: 5'-TGTGGTCATGAGTCCTTCCA-3'	300	58

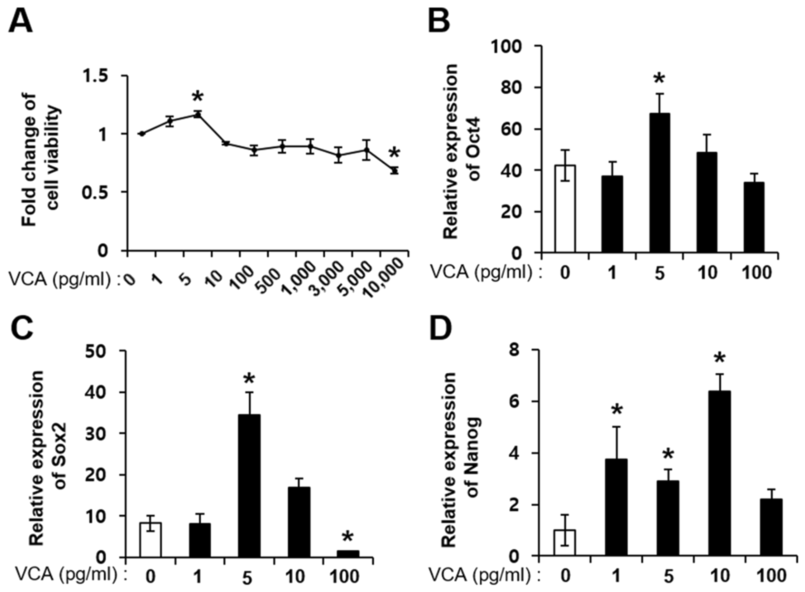

Figure S1. Effect of VCA on the viability and self-renewal of BM-MSCs. (A) Proliferation assay in BM-MSCs at the VCA concentration determined by MTT analysis. Expression of (B) Oct4, (C) Sox2 and (D) Nango in PD-MSCs treated with VCA as determined by western blotting. All reactions were performed in triplicate. Data are shown as the mean ± S.E.; * indicates a significant difference compared to the untreated group (*p<0.05*).

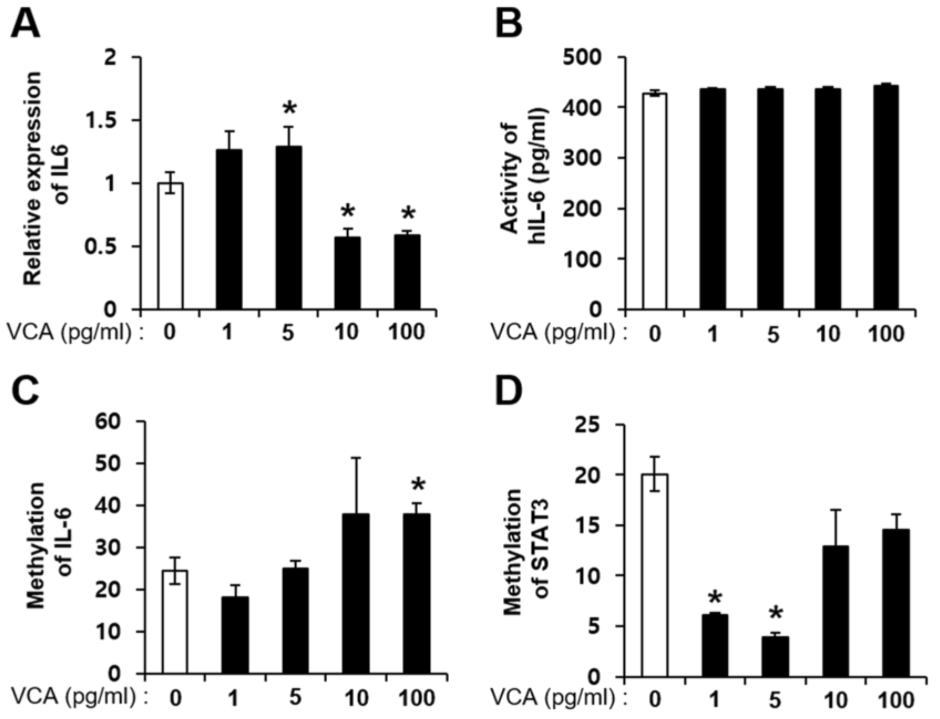

Figure S2. Effect of VCA on the alteration of the IL-6/STAT3 signaling pathway in BM-MSCs. (A) mRNA and (B) protein expression of human IL-6 in BM-MSCs treated with VCA as determined by qRT-PCR. 18S rRNA was used as an internal control. (C) IL-6 and (D) STAT3 were measured by qMSP. The relative quantification of the amplified methylation was determined as the PMR. All reactions were performed in triplicate. Data are shown as the mean ± S.E.; * indicates a significant difference compared to the untreated group (*p<0.05*).
